# Electronic charts do not facilitate the recognition of patient hazards by advanced medical students: A randomized controlled study

**DOI:** 10.1371/journal.pone.0230522

**Published:** 2020-03-26

**Authors:** Friederike Holderried, Anne Herrmann-Werner, Moritz Mahling, Martin Holderried, Reimer Riessen, Stephan Zipfel, Nora Celebi

**Affiliations:** 1 Department of Anaesthesiology, University Hospital Tübingen, Tübingen, Baden-Württemberg, Germany; 2 Department of Internal Medicine VI, Psychosomatic Medicine, University Hospital Tübingen, Baden-Württemberg, Tübingen, Germany; 3 Department of Diabetology, Endocrinology, Nephrology, Section of Nephrology and Hypertension, University Hospital Tübingen, Tübingen, Baden-Württemberg, Germany; 4 Department of Quality Management, Medical and Business Development, University Hospital of Tübingen, Tübingen, Baden-Württemberg, Germany; 5 Department of Internal Medicine VIII, Intensive Care Unit, University Hospital Tübingen, Tübingen, Baden-Württemberg, Germany; 6 PHV Dialysis Center Waiblingen, Waiblingen, Germany; Robert Bosch Krankenhaus, GERMANY

## Abstract

Chart review is an important tool to identify patient hazards. Most advanced medical students perform poorly during chart review but can learn how to identify patient hazards context-independently. Many hospitals have implemented electronic health records, which enhance patient safety but also pose challenges. We investigated whether electronic charts impair advanced medical students’ recognition of patient hazards compared with traditional paper charts. Fifth-year medical students were randomized into two equal groups. Both groups attended a lecture on patient hazards and a training session on handling electronic health records. One group reviewed an electronic chart with 12 standardized patient hazards and then reviewed another case in a paper chart; the other group reviewed the charts in reverse order. The two case scenarios (diabetes and gastrointestinal bleeding) were used as the first and second case equally often. After each case, the students were briefed about the patient safety hazards. In total, 78.5% of the students handed in their notes for evaluation. Two blinded raters independently assessed the number of patient hazards addressed in the students’ notes. For the diabetes case, the students identified a median of 4.0 hazards [25%–75% quantiles (Q25–Q75): 2.0–5.5] in the electronic chart and 5.0 hazards (Q25–Q75: 3.0–6.75) in the paper chart (equivalence testing, p = 0.005). For the gastrointestinal bleeding case, the students identified a median of 5.0 hazards (Q25–Q75: 4.0–6.0) in the electronic chart and 5.0 hazards (Q25–Q75: 3.0–6.0) in the paper chart (equivalence testing, p < 0.001). We detected no improvement between the first case [median 5.0 (Q25–Q75: 3.0–6.0)] and second case [median, 5.0 (Q25–Q75: 3.0–6.0); p < 0.001, test for equivalence]. Electronic charts do not seem to facilitate advanced medical students’ recognition of patient hazards during chart review and may impair expertise formation.

## Introduction

Patient safety incidents are very common and likely to result in avoidable harm, especially diagnostic and prescribing incidents [1]. According to an analysis of malpractice claims by Saber Tehrani et al. [2], diagnostic errors account for 28% of all medical errors and are most likely to lead to permanent disability or death. Most problems arise from history-taking, examination, or failing to order diagnostic tests for further work-up [3].

Other major sources of patient harm are prescribing errors and adverse drug events [4, 5]. The reported incidence of prescription errors among inpatients is about 6% of overall prescriptions, and the incidence of adverse drug events is 19% of admissions; one-third of these adverse drug events are considered preventable [5, 6].

Evidence-based practices to prevent patient harm include preventing infections, implementing measures to reduce medication errors, monitoring patient safety problems and establishing patient safety practices targeted at diagnostic errors, and using checklists [4, 7–9]. In particular, patient chart review promotes patient safety and is a major tool in the recognition of patient hazards such as diagnostic errors, prescription errors, and adverse drug events [10].

Patient charts have traditionally been on paper; however, most hospitals have now adopted electronic health records [11, 12]. With respect to adverse drug events, electronic charts prevent a major source of medication errors: unintelligible abbreviations [4]. Apart from better legibility, electronic health records offer other features that promote patient safety. Evidence-based practices to prevent diagnostic errors include technology-based alerting systems and computerized clinical decision support systems [13–15].

However, there is currently no evidence that electronic patient charts actually reduce adverse drug events [4]. Palojoki et al. [16] even found significantly more patient safety incidents in a fully digitalized environment, and most were attributed to difficulties in human–computer interactions.

One reason for this phenomenon may be that a huge proportion of chart review and prescribing in hospitals is performed by novice physicians [5, 17]. In surveys, advanced medical students have reported a feeling of unpreparedness to manage patients safely [18–21]. According to a cross-sectional study by Pearce et al. [22], the majority of Foundation Year 1 doctors in the UK performed ward rounds alone and unsupervised, and only 7% reported that they felt prepared for this task.

Most patient hazards do not arise from single drugs or diseases, but rather from the context [17, 23]. Practice and experience are required to develop the expertise to manage patients with multiple morbidities [17, 23]. However, studies have shown that with practice and training, advanced students can acquire the skills necessary to identify patient hazards during chart review and to safely and context-independently prescribe medications [24–27]. Significant effects on the detection of patient hazards during chart review were shown even after reviewing a single chart [24].

An integrated adult learning model outlines the following steps in the formation of expertise [28]:

- Dissonance: the learner is confronted with gaps in knowledge or skill
- Refinement and organization: the learner gathers new information and restructures it with the existing body of knowledge
- Feedback: the newly learned information is articulated or tested in new situations
- Consolidation: the learner reflects on the whole learning cycle; e.g., while applying the knowledge in a different context

The bottleneck for the crucial refinement and organization phase is the cognitive load [29, 30]. The cognitive load comprises the internal cognitive load, which is basically the inherent complexity of the task at hand, the extraneous cognitive load, which comprises external factors complicating the task; and the germane load, which is determined by the efficacy of the learner’s processing [29–31].

Revising patient charts is a very complex task that is seldom practiced spontaneously during internships [32–34]. Students usually perform very poorly during simulated chart reviews and ward rounds [24, 34, 35]. Various skills and competencies have been suggested to be implemented in medical schools to promote patient safety, including patient safety curricula and simulation trainings [4, 36–38]. In chart reviews, many of these skills must be applied simultaneously; therefore, knowledge about the patient’s diagnoses, comorbidities, diagnostic and therapeutic procedures, and treatment protocols as well as pharmaceutical knowledge must be integrated with patient safety skills revolving around the avoidance of diagnostic errors and safe prescribing [36]. Chart review thus has a very high intrinsic cognitive load that may limit the refining and organization phase during expertise-building [28, 30]. Case complexity increases the cognitive load, which is reduced by growing knowledge and more advanced illness scripts (a more efficient organization of knowledge with mental “shortcuts” between relevant items of information) [39–43].

However, when handling the electronic health record poses an additional challenge, this may represent an extraneous cognitive load [30]. The increase in cognitive load would further enhance the difficulty of the already challenging task of chart review [29–31, 44]. This might counteract the advantages of electronic charts. Moreover, the increased cognitive load might further interfere with expertise-building [29–31, 44].

In the present study, we investigated whether fifth-year medical students identify more or less patient hazards when a standardized patient case is presented in an electronic chart as opposed to a paper chart and whether the number of recognized patient hazards changes when students review a paper chart after reviewing an electronic chart or vice versa. We hypothesized that the advantages and disadvantages of electronic charts compensate each other in comparison to paper charts and thus checked for equality.

## Methods

### Study design

**Fig 1:**
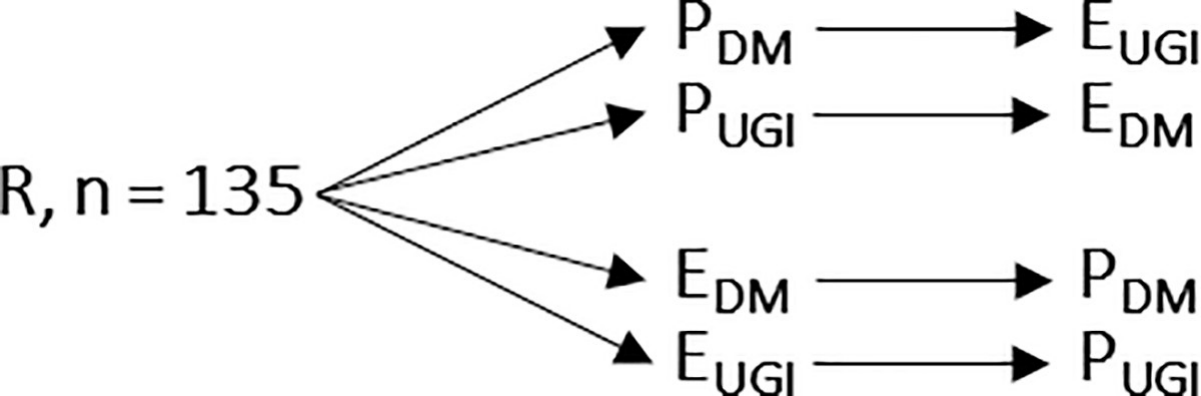
Randomization of study participants. E, electronic chart; P, paper chart; DM, diabetes mellitus case; UGI upper gastrointestinal bleeding case.

In this randomized prospective trial, we used 2 standardized patient cases (diabetes mellitus and upper gastrointestinal bleeding) with 12 standardized patient hazards each. All fifth-year medical students (n = 135) were randomized into two equally sized groups using lots. One group was assigned to review a paper chart first, then review an electronic chart. The other group reviewed an electronic chart first, then reviewed a paper chart. Both groups were further divided into equally sized subgroups so that one half reviewed the diabetes mellitus case first and the upper gastrointestinal bleeding case second, and the other half reviewed the cases in reverse order (Fig 1).

One group reviewed a standardized patient case on a paper chart first, then reviewed the other case on an electronic chart. The other group reviewed a standardized case on an electronic chart first, then reviewed the other case on a paper chart. Each group was divided into two equally sized subgroups so that each assessment case served as the first or second case for half of the group, respectively. In total, 78.5% of the students voluntarily submitted their notes for evaluation.

### Participants and setting

The training and assessment were integrated in the fifth-year practical internal medicine class. Our university has a conventional 6-year curriculum. The fifth year is the last year at the university, during which the students attend classes with formal lectures. The sixth and final year comprises internships at university or teaching hospitals. Thus, the students had already completed their theoretical training in pharmacology and internal medicine. While the course was mandatory, the pseudonymized notes were only evaluated if the students had volunteered them and after the students had provided written consent. The participants were blinded to the study question.

On the first day of the course, all students attended a 45-minute lecture on patient hazards. During this lecture, all of the hazard patterns represented in the standardized charts were discussed extensively. All students then attended a 120-minute standardized instruction on how to operate the electronic health record. This instruction was provided by a trained instructor according to the teaching manual for medical personnel. This training comprised all of the elements suggested by Goveia et al. [45], including classroom training, computer-based training, and feedback.

Next, the students were given questionnaires on their demographic data. The students reviewed the first standardized chart within 30 minutes, ordering diagnostic tests and prescribing medication as they saw fit. We then briefed the students about the expected patient hazards. The second standardized chart was presented on the second day, followed by a briefing of the second standardized chart.

### Electronic chart

We used the electronic health record used in our university hospital (MEONA version 77.551_e; Meona GmbH, Freiburg, Germany). It contains a section in which the diagnoses of the patient are recorded; a page with the patient’s history and physical examination findings; a page with the actual chart including vital signs, medications, and planned diagnostic or therapeutic procedures (chart); a page with laboratory results; and a section on test results such as imaging or endoscopy.

The electronic health record contains highlighted fields in which allergies, general warnings, and infections can be recorded. While prescribing, a patient safety module alerts the prescriber to a known allergy. Information about the drugs, including indications, contraindications, and dosage recommendations, can be accessed via the electronic health record.

### Paper chart

For the paper chart, we used the system that was employed by our university hospital prior to implementation of the electronic health record and that is still used in many German hospitals. It comprises a sheet summarizing the diagnoses, history, physical examination findings, and test results (i.e., imaging, endoscopy); the main chart, which contains vital signs, medications, and diagnostic tests and that is both used by nursing staff and doctors; and a separate file for laboratory results.

The students were given paper-based prescription aids listing drug indications, contraindications, and dosage recommendations when reviewing the paper patient charts.

### Assessment

For the assessment, 2 fictional patient cases were constructed with 12 standardized common patient hazards:

- One indicated medication is missing
- One medication is not indicated
- One medication has the incorrect dosage
- One risk situation is present for an unauthorized medication
- One medication has adverse effects
- One medication is contraindicated
- One incidental actionable diagnostic finding is present
- One diagnostic test for the main problem is missing
- The monitoring for the main problem is incomplete
- One infectious complication is present
- Diet/fluid management is incorrect
- The documentation is incomplete

Both cases were presented either as a paper chart or as an electronic chart containing identical information. The cases were developed by two physicians and reviewed by two other physicians to ensure face validity. An example of an assessment case is shown in **Table 1**.

**Table 1:** Example of an assessment case with standardized patient hazards.

HAZARD	UPPER GASTROINTESTINAL BLEEDING
**INDICATED MEDICATION MISSING**	Long-acting beta-mimetic for comorbidity (chronic obstructive pulmonary disease)
**MEDICATION NOT INDICATED**	Allopurinol
**INCORRECT DOSAGE**	Tiotropium bromide
**RISK SITUATION FOR UNAUTHORIZED MEDICATION**	Pain for knee osteoarthritis
**ADVERSE EFFECT**	Diclofenac (upper gastrointestinal bleeding)
**CONTRAINDICATION**	Allergy to contrast material
**INCIDENTAL ACTIONABLE DIAGNOSTIC FINDING**	Hyperthyroidism
**DIAGNOSTIC TEST MISSING**	Biopsy/computed tomography/endosonography
**MONITORING INCOMPLETE**	Blood count
**INFECTIOUS COMPLICATION**	Infected central line
**INCORRECT DIET/FLUID MANAGEMENT**	Gluten-free diet in celiac disease
**DOCUMENTATION INCOMPLETE**	Hyperthyroidism, gastric tumor

The participants marked their charts with pseudonyms comprising the first letter of the mother’s given name, the first letter of the place of birth, the second letter of the own given name, the first letter of the month of birth, and the day of birth.

After completion of the assessment, the paper charts were then transferred into an electronic chart, and both the electronic and paper charts were printed so that they were indistinguishable to the raters. The charts were sorted alphabetically according to the pseudonyms.

Two blinded raters assessed the charts independently according to predefined rating criteria using a checklist. One rater rated them from the top down and the other rated them from the bottom up to minimize observer drift. Whenever one patient hazard was addressed in the student’s prescription, the raters awarded 1 point irrespective of whether the reaction was adequate. During the rating process, both raters noted when predefined rating criteria were unclear for a specific chart; the raters then discussed and redefined the rating criteria and rerated the disputable charts independently.

### Statistics

Statistical analyses were performed using R [1], and graphics were drawn using Prism Version 7.0e (GraphPad Software, San Diego, CA, USA). We assumed a Gaussian distribution because of the large sample size and numeric rating scores. Data are described as median and 25%–75% quantiles (Q25–Q75) and presented as box plots (Tukey whiskers).

To assess the inter-rater reliability, we calculated the intraclass correlation coefficient (ICC) using R [1] and the irr package [2]. We assessed the total checklist score for consistency using a one-way model (ICC type “3,1” according to Shrout and Fleiss [46]).

We planned to pool data from the first and second case that the students reviewed and thus assumed them to be independent because each participant had a change in presentation (paper vs. electronic) as well as disease (diabetes mellitus vs. upper gastrointestinal bleeding). We therefore performed an effect screening using least squares and the following potential influences: time (first case vs. second case), presentation (paper vs. electronic), and disease (diabetes mellitus vs. upper gastrointestinal bleeding).

Our intention was to test for statistical equivalence instead of statistical differences. We assumed that a score difference of 2 out of 12 recognized hazards as clinically equivalent (i.e., a clinically insignificant difference). We performed a retrospective power analysis and equivalence testing using two one-sided tests (TOST) equivalence testing [1].

### Ethics

The institutional review board (Ethik-Kommission an der Medizinischen Fakultät der Eberhard-Karls-Universität und am Universitätsklinikum Tübingen) approved this study (decision number 2602016BO2). The need for detailed review by the board was waived since no patients were involved and study participation for the students was voluntary and pseudonymized. We informed the students at the beginning of the study about the voluntary nature of their participation and obtained written informed consent nonetheless. The students could decline to provide consent at any given time without giving a reason and without disadvantages for the course.

## Results

### Demographic data

Of all 135 fifth-year medical students, 106 (78.5%) voluntarily submitted their notes for evaluation. These students’ demographic data are presented in **Table 2**.

**Table 2:** Demographic data of students who submitted their notes on the standardized patient charts for evaluation.

	DIABETES MELLITUS CASE: ELECTRONIC CHART	DIABETES MELLITUS CASE: PAPER CHART	UPPER GASTROINTESTINAL BLEEDING CASE: ELECTRONIC CHART	UPPER GASTROINTESTINAL BLEEDING CASE: PAPER CHART
**N**	55	47	47	55
**AGE, YEARS**	26.3 ± 3.4	25.4 ± 3.1	25.4 ± 3.1	26.3 ± 3.4
**SEMESTER**	9.0 ± 0.2	9.1 ± 0.2	9.1 ± 0.2	9.0 ± 0.2
**FEMALE SEX**	28 (62)	24 (65)	24 (65)	28 (62)
**COMPLETED PROFESSIONAL TRAINING PRIOR TO MEDICAL SCHOOL**	13 (29) 3 nurses, 8 paramedics, 2 not specified	14 (39) 4 nurses, 7 paramedics, 3 not specified	14 (39) 4 nurses, 7 paramedics, 3 not specified	13 (29) 3 nurses, 8 paramedics, 2 not specified
**HAS REVIEWED PATIENT CHARTS IN THE PAST**	29 (64)	22 (61)	22 (61)	29 (64)
**HAS USED AN ELECTRONIC CHART SYSTEM IN THE PAST**	29 (66)	20 (57)	20 (57)	29 (66)

Data are presented as mean ± standard deviation or n (%).

### Power analysis and inter-rater reliability

The retrospective power analysis for equivalence testing resulted in a power of 0.999.

The inter-rater reliability assessed using the ICC was 0.997 (0.996–0.998) for the upper gastrointestinal bleeding case and 0.988 (0.982–0.992) for the diabetes mellitus case, indicating excellent inter-rater reliability for both charts [3].

### Screening for effects

We screened time (first case vs. second case), presentation (paper vs. electronic), and diseases (diabetes mellitus vs. upper gastrointestinal bleeding) with respect to their influence on the number of recognized hazards. None of the screened effects displayed a significant impact on the number of recognized hazards. We therefore pooled data from the first and second case presentations (i.e., electronic chart and diabetes mellitus as the first case and electronic chart and diabetes mellitus as the second case).

### Effect of presentation as electronic or paper chart on recognized patient hazards

**Fig 2:**
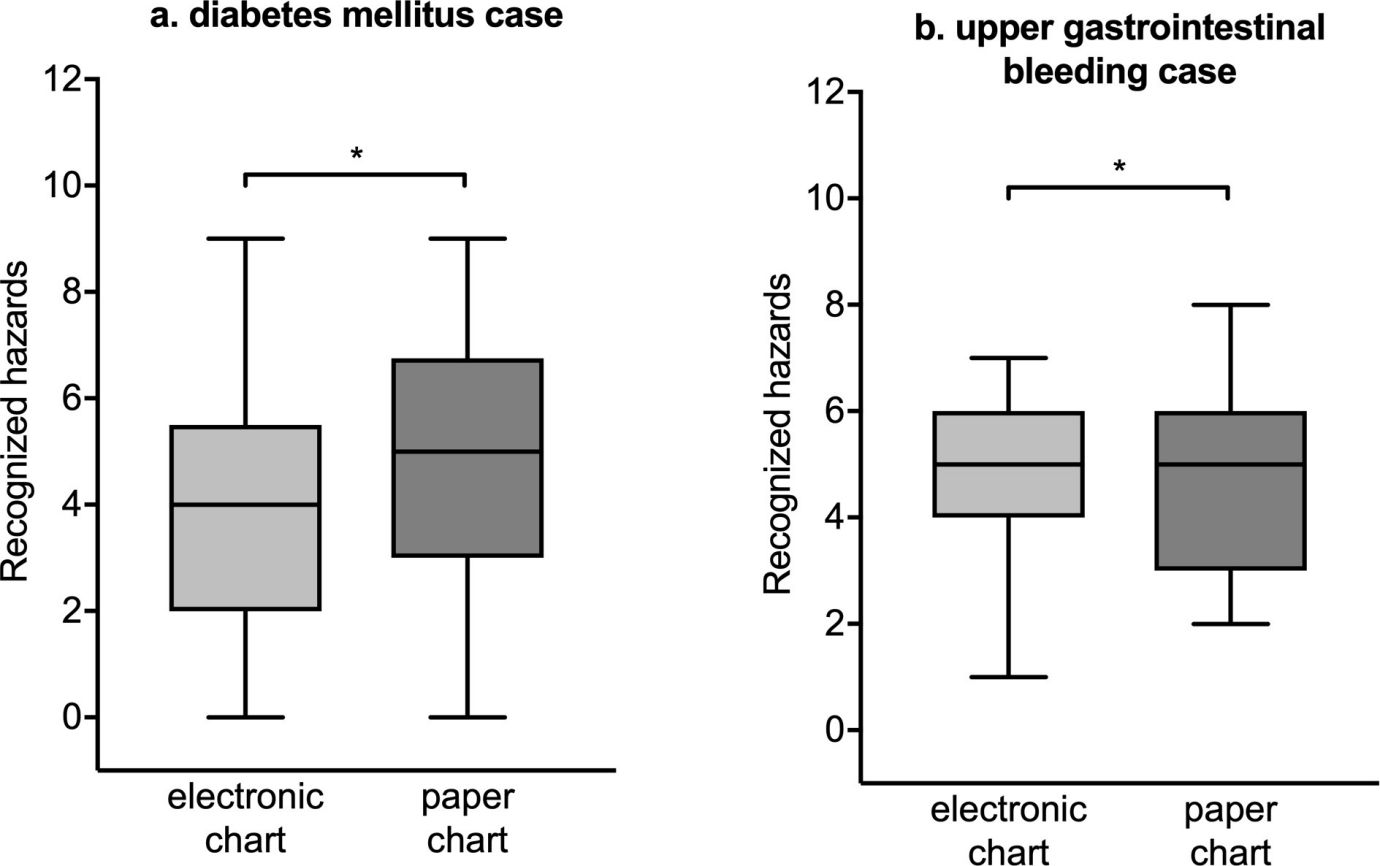
Number of recognized patient hazards. (a) Diabetes mellitus case. (b) Upper gastrointestinal bleeding case. Box plots (Tukey whiskers) for presentation as electronic chart or paper chart. *p < 0.05 for statistical equivalence.

The number of recognized patient hazards for electronic charts versus paper charts is shown in Fig 2. For diabetes mellitus (Fig 2A), the participants recognized a median of 4.0 hazards (Q25–Q75: 2.0–5.5) in the electronic chart and a median of 5.0 hazards (Q25–Q75: 3.0–6.75) in the paper chart (equivalence testing, p = 0.005, indicating statistically equivalent groups). When we presented the upper gastrointestinal bleeding case to the participants, they recognized a median of 5.0 hazards (Q25–Q75: 4.0–6.0) in the electronic chart and a median of 5.0 hazards (Q25–Q75: 3.0–6.0) in the paper chart (equivalence testing, p < 0.001, indicating statistically equivalent groups).

**Fig 3:**
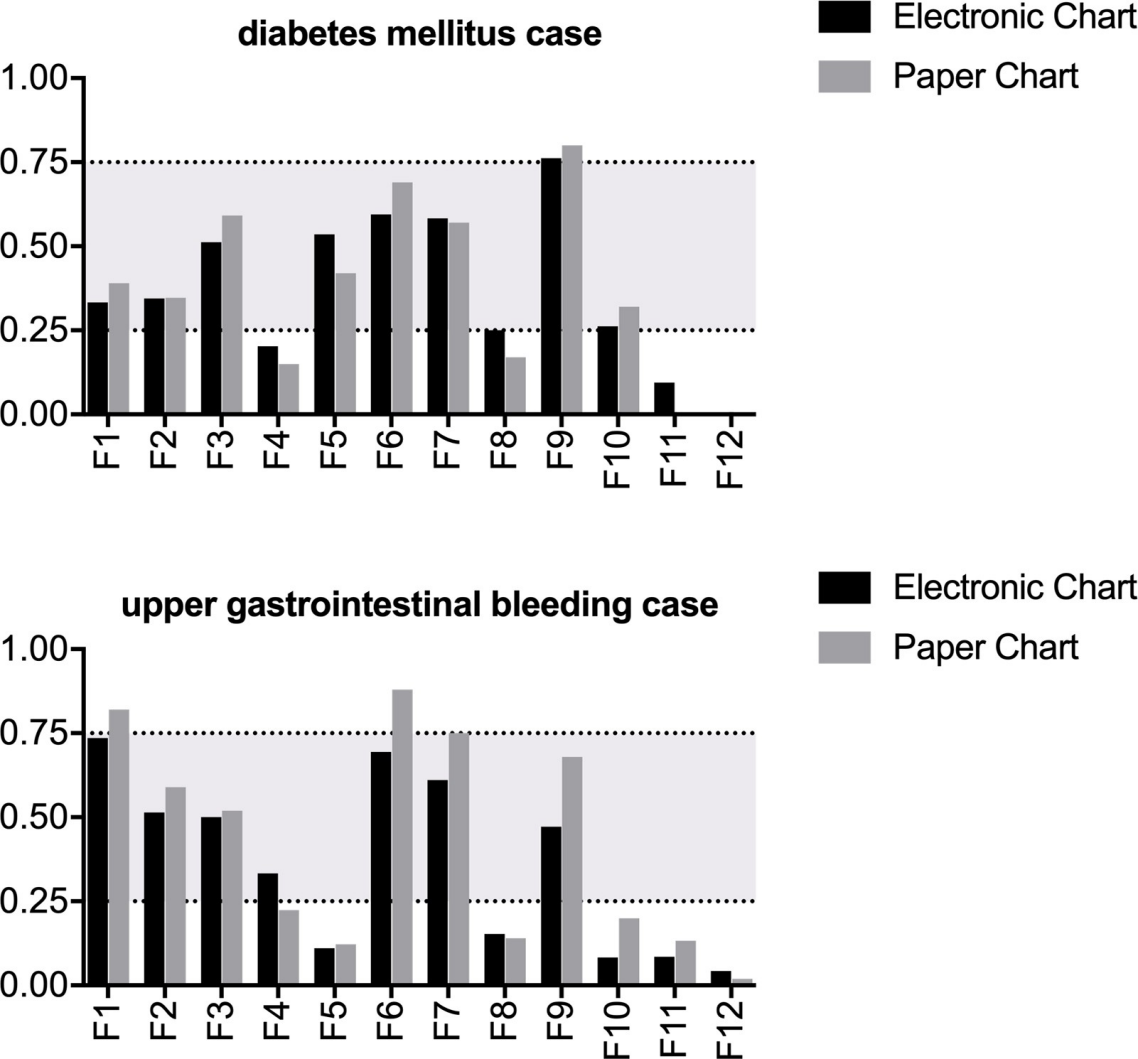
Type of recognized patient hazard by case and chart. F1: One diagnostic test for the main problem is missing, F2: One incidental actionable diagnostic finding is present, F3: One medication is contraindicated, F4: One medication has the incorrect dosage, F5 One indicated medication is missing, F6 One medication has adverse effects, F7: One medication is not indicated, F8: One infectious complication is present, F9: The monitoring for the main problem is incomplete, F10: Diet/fluid management is incorrect, F 11 The documentation is incomplete, F12 One risk situation is present for an unauthorized medication.

In Fig 3, we display the types of patient hazards recognized by chart.

### Transition between electronic chart and paper chart

A total of 77 students handed in both cases for evaluation. For these students, we tested whether an improvement had occurred between the first and second case.

**Fig 4:**
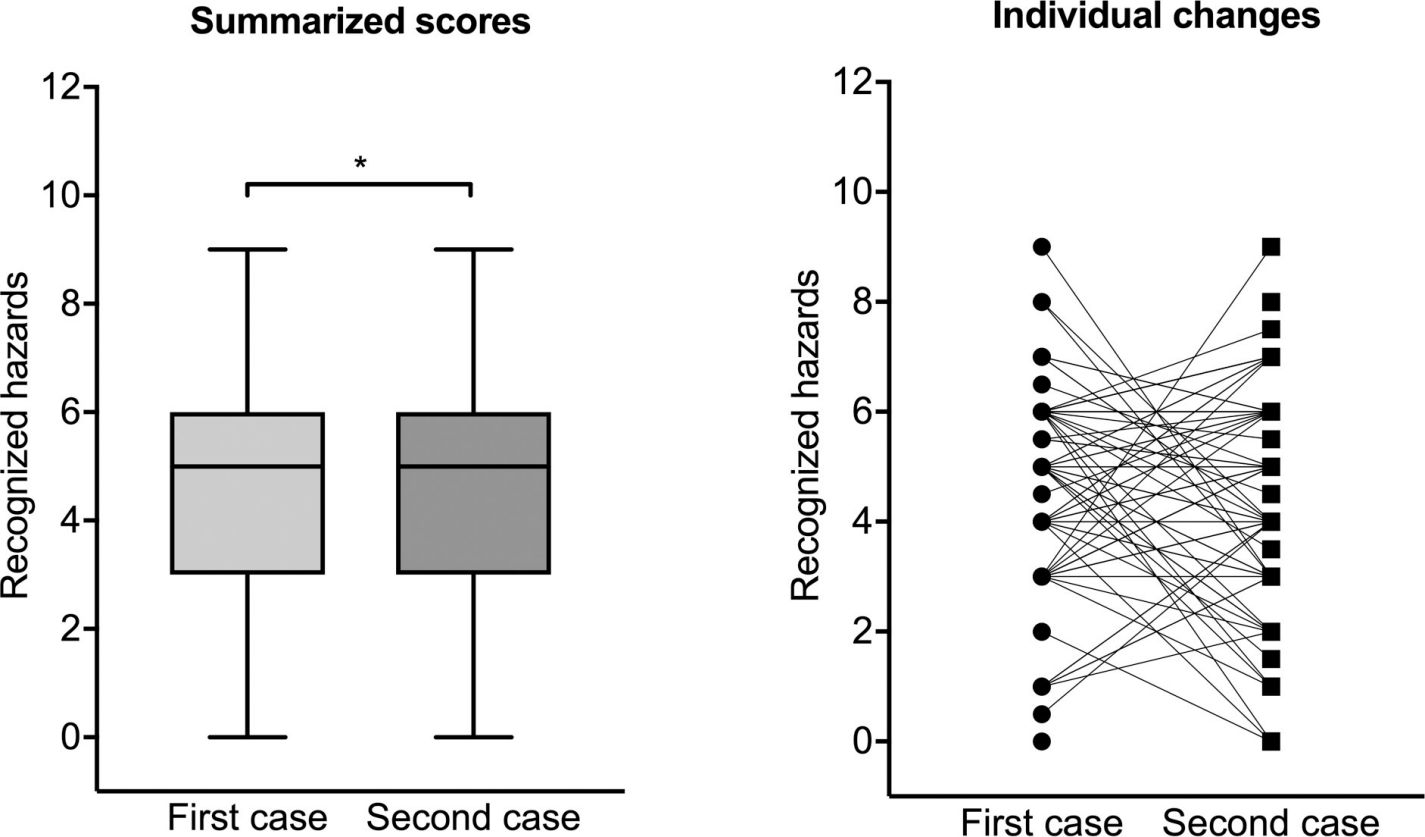
Overall recognition of patient hazards when presented in different charts (electronic or paper). There was no measurable improvement in the second case despite an extensive briefing after the first case. *p < 0.05 for statistical equivalence.

Overall, we detected no improvement when the students reviewed the second case presented in a different chart, either electronic or paper [median: 5.0 (Q25–Q75: 3.0–6.0) in the first case vs. median: 5.0 (Q25–Q75: 3.0–6.0) in the second case, p < 0.001, test for equivalence] (Fig 4).

**Fig 5:**
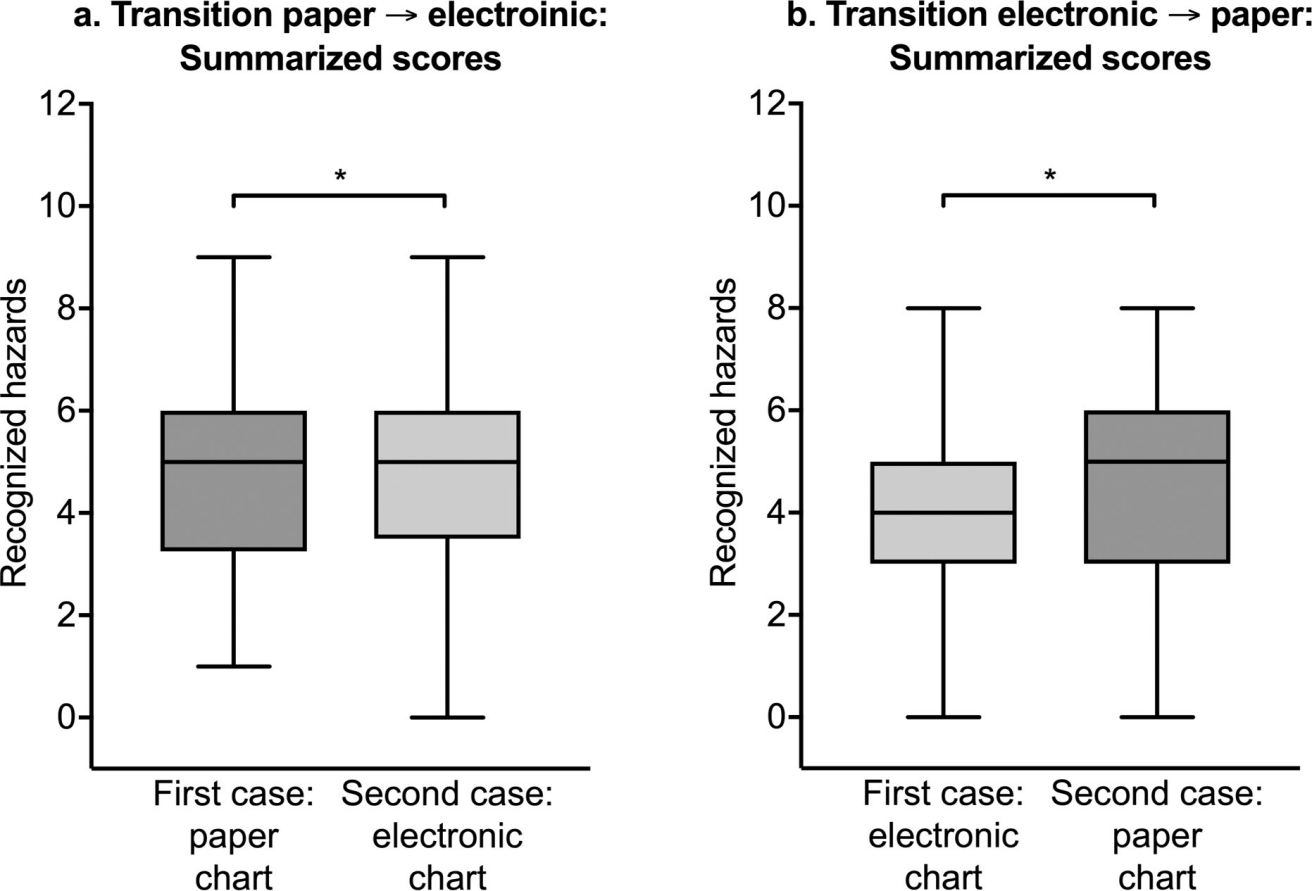
Numbers of patient hazards recognized by fifth-year medical students. (a) Transitioning from a paper chart to another case presented in an electronic chart. (b) Transitioning from an electronic chart to another case presented in a paper chart. *p < 0.05 for statistical equivalence.

When transitioning from a paper chart to an electronic chart, the students recognized a median of 5.0 hazards (Q25–Q75: 3.25–6.0) versus 5.0 hazards (Q25–Q75: 3.5–6.0) (p < 0.001 for statistical equivalence). When transitioning from an electronic chart to a paper chart, the students recognized a median of 4.0 hazards (Q25–Q75: 3.0–5.0) versus 5.0 hazards (Q25–Q75: 3.0–6.0) (p < 0.001 for statistical equivalence) (Fig 5).

## Discussion

In this study, we investigated whether electronic patient charts facilitate the recognition of patient hazards during chart reviews by advanced medical students. We found that the students did not recognize more patient hazards when the data were presented in an electronic chart as opposed to a paper chart. Moreover, we detected no improvement when the students reviewed another case presented in the other medium despite the fact that the students had received a lecture on which patient hazards to expect in advance and had received an extensive briefing after each case. In our previous study with a similar methodology, the students were presented three different case scenarios on a paper chart, and the students identified significantly more patient hazards with every additional case presented [24]. The number of recognized patient hazards was comparable to the baseline finding in our previous study, we attributed the low scoring to the fact that chart review is only seldomly practiced during clerkships and for most of the students this was the first chart review ever they performed [24, 34].

Despite the advantages that electronic health records confer in comparison to paper charts, such as legibility and electronic alert flags or decision-making tools, Palojoki et al. [4, 16] actually found more patient safety incidents in a fully electronic environment than in historical controls. In Japanese hospitals, productivity decreased with the implementation of electronic health records [47]. This may be partially explained by usability and navigation issues [48, 49]. According to a study by Kaipio et al. [50], usability of electronic health records was rated poorly in Finland and did not improve from 2010 to 2014. A study by Clarke et al. [51] showed no relevant difference in task completion amongst expert and novice users of electronic health records, indicating that usability problems may persist even after longer exposure to the electronic health record. However, another study showed improvements in the use of electronic health records with prolonged exposure [52].

If usability and navigation issues with the electronic health record had been the only problem, we would have expected to detect an improvement in the recognition of patient hazards in the transition from the electronic chart to the paper chart and stagnation with transition from the paper chart to the electronic chart. However, we found no improvement in either direction despite an extensive briefing between the two cases. There are indications that chart review on paper is partially perceived as a different task than chart review with an electronic health record. In their qualitative study, Borycki et al. [53] found that nursing students sought different information when presented a case on a paper chart versus on a hybrid paper/electronic chart. Additionally, physicians documented different physical examination findings in a paper-based record than in an electronic health record [54]. Skills such as safe prescribing and the diagnostic process can be partially taught in a context-independent manner [55, 56]. This requires the transfer of concepts, which is usually very difficult and requires several examples; the closer the cases are, the easier the transfer is achieved [57–60]. Thus, when a transfer between media is required in addition to the transfer between contexts, this might increase the cognitive load, impair transfer, and thus impair expertise formation. This might explain why there was no measurable improvement between the two cases in comparison to our previous study when all cases were presented in the same, paper-based format. In our study, there was a difference in the recognized patient hazards, the students identified more adverse effects of drugs in the paper chart than in the electronic health record in both cases. However, difficulty of the cases is highly context-dependent, so more cases and scenarios would have to be analyzed before drawing a conclusion.

Our study has several limitations. First, it was a small monocentric study investigating the performance of one cohort of medical students only. We did not directly measure the cognitive load but instead relied on measurement of the number of recognized patient hazards.

Because the students handed in their notes for evaluation on a voluntary basis, we cannot exclude sampling bias despite the fact that the majority of the cohort participated. However, we would expect a distortion of the data toward better performance under the assumption that the more confident students would hand in their notes.

Because all students were briefed about which patient hazards were to be expected, we do not assume that our findings were attributable to a lack of knowledge, although we cannot exclude this possibility. Another alternative explanation might be a ceiling effect; however, because the students identified only an average of 4 to 5 out of 12 patient hazards, we assume that this is unlikely.

Although the students were trained for 120 minutes on the handling of the electronic patient records, including documentation and prescribing exercises, usability problems might have led to underperformance when reviewing the electronic chart.

Future studies should be performed to assess whether our findings can be replicated with a larger number of cases, in different contexts, and with different electronic health records and to determine how many cases are needed to improve the recognition of patient hazards when presented with an electronic health record. When developing or purchasing an electronic health record, responsible persons should pay attention to usability in order to facilitate the recognition of patient hazards, and when designing a curriculum on patient hazards and chart review, responsible persons should bear in mind that this difficult skill has to be practiced, especially when using an electronic health record.

## Conclusion

Electronic charts do not seem to facilitate advanced medical students’ identification of patient hazards compared with paper charts and probably interfere with expertise building.

## Supporting information

S1 Dataset Original dataset for data availability. (XLSX)
